# SOX30 Facilitates Triple‐Negative Breast Cancer Metastasis via TNFR2–NF‐κB Signaling and Tumor Microenvironment Remodeling

**DOI:** 10.1002/advs.77690

**Published:** 2026-09-09

**Authors:** Pingping Gao, Na Sun, Ning Yang, Tingting Zhao, Rui Fu, Zhenyu Wu, Yuqin Zhou, Xuanni Tan, Yi Zhang, Fei Han, Xiaowei Qi

**Affiliations:** ^1^ Department of Breast and Thyroid Surgery, Southwest Hospital Army Medical University Chongqing People's Republic of China; ^2^ Key Laboratory of Chongqing Health Commission for Minimally Invasive and Precise Diagnosis Chongqing People's Republic of China; ^3^ Department of Toxicology, School of Public Health Chongqing Medical University Chongqing People's Republic of China; ^4^ Joint International Research Laboratory of Reproduction and Development of the Ministry of Education Chongqing People's Republic of China

**Keywords:** metastasis, SOX30, triple‐negative breast cancer, tumor microenvironment, tumor‐associated macrophages

## Abstract

Triple‐negative breast cancer (TNBC) remains therapeutically challenging due to its aggressive metastatic potential, high recurrence rates, and lack of effective targeted therapies. Herein, we identified that SOX30 is significantly upregulated in TNBC and correlates with poor clinical prognosis. Functionally, SOX30 specifically drives TNBC metastasis and activates the TNFR2‐mediated downstream NF‐κB signaling cascade. Mass cytometry profiling of the tumor immune microenvironment revealed that SOX30 facilitates the recruitment of tumor‐associated macrophages (TAMs) within pulmonary metastases, a finding corroborated by flow cytometric quantification. Mechanistically, downstream pathway analyses identified that SOX30 induces activation of the TNFR2–NF‐κB axis and concurrently upregulates expression of the chemokine CCL20. Therapeutic intervention with CCL20 neutralizing antibodies or small‐molecule CCR6 inhibitors effectively attenuated metastatic progression and abrogated TAM infiltration. Strikingly, combinatorial blockade using the TNFR2 inhibitor etanercept together with a CCR6 inhibitor exhibited superior anti‐metastatic efficacy relative to control treatments. Collectively, our findings delineate a novel oncogenic SOX30–TNFR2–NF‐κB–CCL20/CCR6 axis driving TNBC metastasis. These results establish SOX30 as a promising prognostic biomarker and a potential therapeutic target for anti‐metastatic strategies in TNBC.

## Graphical abstract

SOX30 drives triple‐negative breast cancer (TNBC) metastasis through a dual mechanism: direct activation of the TNFR2/NF‐κB signaling cascade and subsequent CCL20‐mediated recruitment of tumor‐associated macrophages (TAMs) into the tumor microenvironment. Combined pharmacological blockade of both the CCL20–CCR6 axis and TNFR2 synergistically and significantly suppresses metastasis in vivo, thereby revealing a highly promising dual‐targeted therapeutic strategy for TNBC.

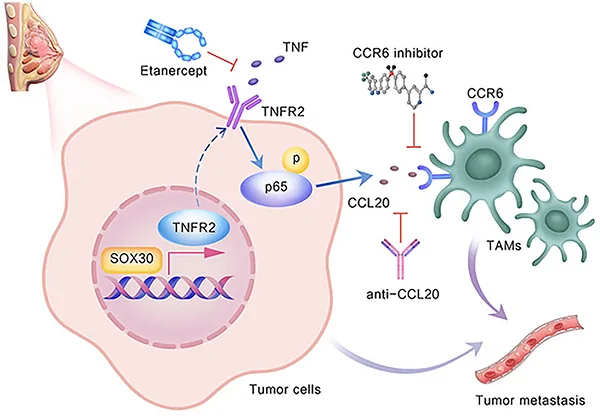

## 1 Introduction

Breast cancer (BRCA) is the most prevalent malignancy among women worldwide [1, 2]. Compared with other subtypes, triple‐negative breast cancer (TNBC) exhibits more aggressive biological behavior, characterized by higher histological grade, poorer clinical outcomes, and an increased risk of early recurrence and distant metastasis [3]. Metastatic progression remains the primary cause of death in TNBC patients [4]. The metastatic cascade involves a series of complex molecular and cellular events that are not yet fully elucidated. Given the limited therapeutic options and poor prognosis of TNBC, uncovering the molecular mechanisms driving its metastasis and identifying novel therapeutic targets are critical for developing more effective treatment strategies and improving outcomes in this high‐risk population.

The SOX family of transcription factors, defined by a conserved high‐mobility group (HMG) DNA‐binding domain, plays a pivotal role in cell fate determination and differentiation during development and in cancer, particularly in tumorigenesis and metastasis [5]. SOX30, a recently characterized SOX family member, was initially identified in zebrafish through comparative genomic analyses [6]. Subsequent functional studies in multiple species, including mice, Nile tilapia, and carp, have established its essential role in testicular development and spermatogenesis [7, 8, 9, 10]. Although SOX30 was initially recognized for its role in reproductive biology, accumulating evidence has implicated it in tumorigenesis, particularly in lung adenocarcinoma, ovarian cancer, and bladder cancer [11, 12, 13, 14, 15]. However, its function in breast cancer remains poorly understood.

The tumor immune microenvironment plays a pivotal role in tumor progression, immune evasion, and therapeutic response [16]. Macrophages represent the predominant population of tumor‐infiltrating immune cells and exist in two main phenotypes: classically activated M1 macrophages, which exert antitumor effects, and alternatively activated M2 macrophages, which typically promote tumor progression [17]. Tumor‐associated macrophages (TAMs) predominantly exhibit an M2‐like polarization state and facilitate cancer metastasis through dynamic interactions with tumor cells [18]. Members of the SOX family have been implicated in modulating the tumor immune microenvironment, including macrophage repolarization [19]. However, the role of SOX30 in macrophage regulation remains unexplored.

In this study, we investigated the role of SOX30 in breast cancer progression, with a specific focus on TNBC. Our findings reveal that SOX30 drives TNBC metastasis through a dual mechanism: direct activation of the TNFR2/NF‐κB signaling cascade and subsequent CCL20‐mediated recruitment of TAMs into the tumor microenvironment. Collectively, these findings enhance the understanding of the molecular mechanisms underlying TNBC metastasis and highlight SOX30 as a potential target for novel therapeutic strategies.

## 2 Results

### 2.1 Expression Levels and Prognosis Relevance of SOX30 in BRCA

**FIGURE 1:**
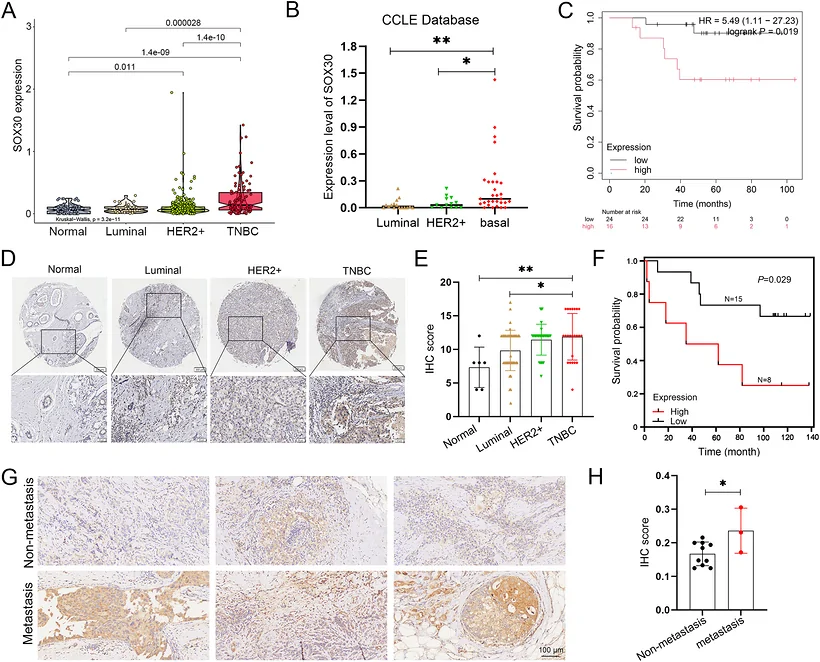
The expression levels and prognostic significances of SOX30 in TNBC. (A) The expression level of SOX30 in different subtypes of breast cancer was analyzed by TCGA database. (B) The expression level of SOX30 in different cell subtypes of breast cancer was analyzed by CCLE database. Normality of data was assessed using the Shapiro–Wilk test. Since the data were not normally distributed, the Kruskal–Wallis test was performed to compare differences among groups, followed by Dunn's post‐hoc test for pairwise comparisons. (C) The survival analysis of patients stratified by SOX30 expression in the TNBC subtype was performed by the Kaplan–Meier plotter database. (D) Immunohistochemistry staining of SOX30 on tissue microarray in breast cancer tissue and paracancer tissue. (E) Protein expression levels of SOX30 in different subtypes of breast cancer. Normality of data was assessed using the Shapiro–Wilk test. Since the data were not normally distributed, the Kruskal–Wallis test was performed to compare differences among groups, followed by Dunn's post‐hoc test for pairwise comparisons. (F) The survival of SOX30 in TNBC patients. (G) Representative images of IHC staining with SOX30 in metastasis and non‐metastasis TNBC patients. Bar = 100 µm. (H) Expression level of SOX30 in metastasis and non‐metastasis TNBC patients. ^*^: *p* < 0.05. ^**^: *p* < 0.01.

To investigate the potential role of SOX30 in breast cancer, particularly in TNBC, immunohistochemistry was used to assess its expression profile and prognostic significance. Analysis of data from the TCGA database revealed that SOX30 was significantly overexpressed in TNBC tissues compared to normal breast tissue, as well as to luminal and HER2‐positive subtypes (Figure 1A). This expression pattern was corroborated at the cellular level using the Cancer Cell Line Encyclopedia (CCLE) database, demonstrating significantly elevated SOX30 mRNA levels in TNBC cell lines relative to those of the luminal and HER2‐positive subtypes (Figure 1B). Kaplan–Meier survival analysis demonstrated that TNBC patients with high SOX30 expression exhibited significantly shorter overall survival compared to those with low expression (Figure 1C), suggesting that SOX30 may function as an independent adverse prognostic indicator in TNBC.

To confirm these findings at the protein level, we performed immunohistochemical (IHC) analysis on a tissue microarray containing 153 tumor specimens and 10 matched adjacent normal tissues. Utilizing a semi‐quantitative scoring system that incorporated both staining intensity and the percentage of positively stained cells, we observed significantly higher SOX30 protein expression in TNBC tissues compared to normal controls (Figure 1D,E). Moreover, elevated SOX30 expression was associated with poorer clinical outcomes in TNBC patients (Figure 1F). Clinicopathological correlation analysis revealed statistically significant associations between high SOX30 expression and clinical stage (p = 0.041), progesterone receptor (PR) status (p = 0.001), and human epidermal growth factor receptor 2 (HER2) status (p = 0.006) (Table 1). However, no significant correlations were observed between SOX30 expression and tumor size, patient age, lymph node status, or tumor location (Table 1). Moreover, the expression of SOX30 was higher in metastatic TNBC compared to that in primary tumors (Figure 1G,H), suggesting an ongoing contribution to the progression of metastasis. These multidimensional findings suggest that SOX30 is not only aberrantly expressed in TNBC but also functionally relevant to its pathogenesis and progression, highlighting its potential as a molecular biomarker in this aggressive subtype of breast cancer.

**TABLE 1:** SOX30 expression correlates with clinicopathologic features of breast cancer patients.

		SOX30 Expression		
Clinical Feature	Total	High (n = 98)	Low (n = 55)	*p* value
Age (years)				
< 52	78	49	29	0.746
≥ 52	75	49	26	
Grade				
1	17	10	7	0.634
2+3	136	88	48	
Stage				
I+II	101	60	41	0.041
III	47	36	11	
Tumor size				
≤ 3 cm	113	72	41	0.951
> 3 cm	38	24	14	
Lymph node				
Negative	57	38	19	0.680
Positive	90	57	33	
Tumor location				
Right	86	55	31	0.977
Left	67	43	24	
ER expression				
Negative	48	36	12	0.065
Positive	104	62	42	
PR expression				
Negative	60	48	12	0.001
Positive	90	48	42	
HER2 expression				
Negative	101	57	44	0.006
Positive	52	41	11	

### 2.2 SOX30 Promotes Tumor Metastasis in TNBC

**FIGURE 2:**
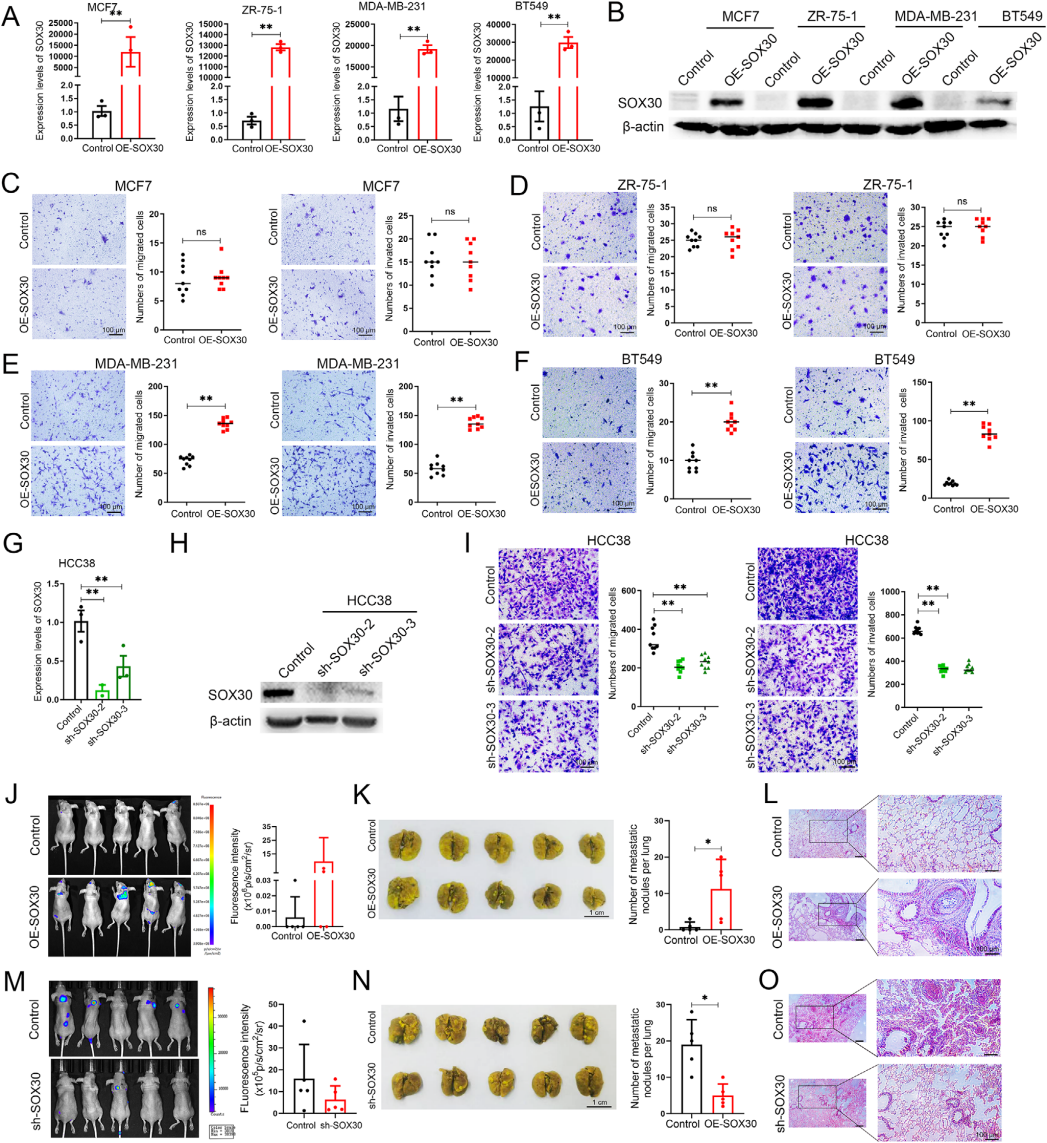
SOX30 facilitates the metastasis of TNBC. (A) Overexpression of SOX30 was detected in MDA‐MB‐231, BT549, MCF7, and ZR‐75‐1 cells by qRT‐PCR. (B) Overexpression of SOX30 was detected in MDA‐MB‐231, BT549, MCF7, and ZR‐75‐1 cells by western blotting. (C) Effects of SOX30 on migration and invasion were performed in MCF7. Bar = 100 µm. (D) Effects of SOX30 on migration and invasion were performed in ZR‐75‐1 cells. Bar = 100 µm. (E) Effects of SOX30 on migration and invasion were performed in MDA‐MB‐231. Bar = 100 µm. (F) Effects of SOX30 on migration and invasion were performed in BT549 cells. Bar = 100 µm. (G) Knockdown of SOX30 was detected in HCC38 cells by qRT‐PCR. (H) Knockdown of SOX30 was detected in HCC38 cells by western blotting. (I) Effects of SOX30 on migration and invasion were performed in HCC38 cells. Bar = 100 µm. (J) Representative fluorescence images and GFP fluorescence intensity of tumors in nude mice injected with SOX30‐overexpressing cells. (K) Images of lung metastasis and statistical graphs of nodules in the SOX30‐overexpressing group and control group. Bar = 1 cm. (L) Image of H&E staining in the SOX30‐overexpressing group and control group. Bar = 1 cm. (M) Representative fluorescence images and GFP fluorescence intensity of tumors in nude mice injected with SOX30‐knockdown cells. (N) Images of lung metastasis and statistical graphs of nodules in the SOX30‐knockdown group and control group. Bar = 1 cm (O) Image of H&E staining in the SOX30‐knockdown group and control group. Bar = 100 µm. ^*^: *p* < 0.05. ^*^: *p* < 0.01.

To investigate the functional role of SOX30 in BRCA, we assessed SOX30 expression levels across various breast cancer cell lines using qRT‐PCR (Figure S1A). Based on these results, we selected two non‐TNBC cell lines (MCF7 and ZR‐75‐1) and two TNBC cell lines (MDA‐MB‐231 and BT549) with relatively low endogenous SOX30 expression for functional studies. Successful overexpression was confirmed at both mRNA and protein levels through qRT‐PCR and western blotting analyses (Figures 2A,B and S1B). MTS proliferation assays revealed that SOX30 overexpression did not significantly affect cell proliferation in either TNBC or non‐TNBC cell lines (Figure S1C). However, SOX30 overexpression significantly enhanced the migration and invasion capacities of TNBC cells (MDA‐MB‐231 and BT549), while still exerting no measurable effect on non‐TNBC cells (MCF7 and ZR‐75‐1) (Figure 2C–F). These results suggest that SOX30 specifically promotes metastatic progression in TNBC.

To further validate the role of SOX30 in TNBC metastasis, rescue experiments were performed by knocking down SOX30 in SOX30‐overexpressing MDA‐MB‐231, HCC38, and HCC1937 cells. Efficient knockdown was confirmed at both the mRNA and protein levels via qRT‐PCR and western blotting (Figures 2G,H and S2A,B). Functional assays revealed that SOX30 depletion did not significantly alter cellular proliferation, as measured by the MTS assay (Figure S1B), but markedly reduced the migratory and invasive capacities of TNBC cells (Figures 2I and S2C,D).

To assess the in vivo relevance of these findings, experimental metastasis models were established by intravenous injection of tumor cells into BALB/c nude mice. Mice injected with SOX30‐overexpressing MDA‐MB‐231 cells developed significantly more lung metastases than control mice at 7 weeks post‐injection (Figure 2J–L). In contrast, mice injected with HCC38 cells stably depleted of SOX30 exhibited a marked reduction in the number of pulmonary metastatic nodules compared to controls (Figure 2M–O).

### 2.3 SOX30 Drives TNBC Metastasis via Activation of TNFR2‐Mediated NF‐κB Pathway

**FIGURE 3:**
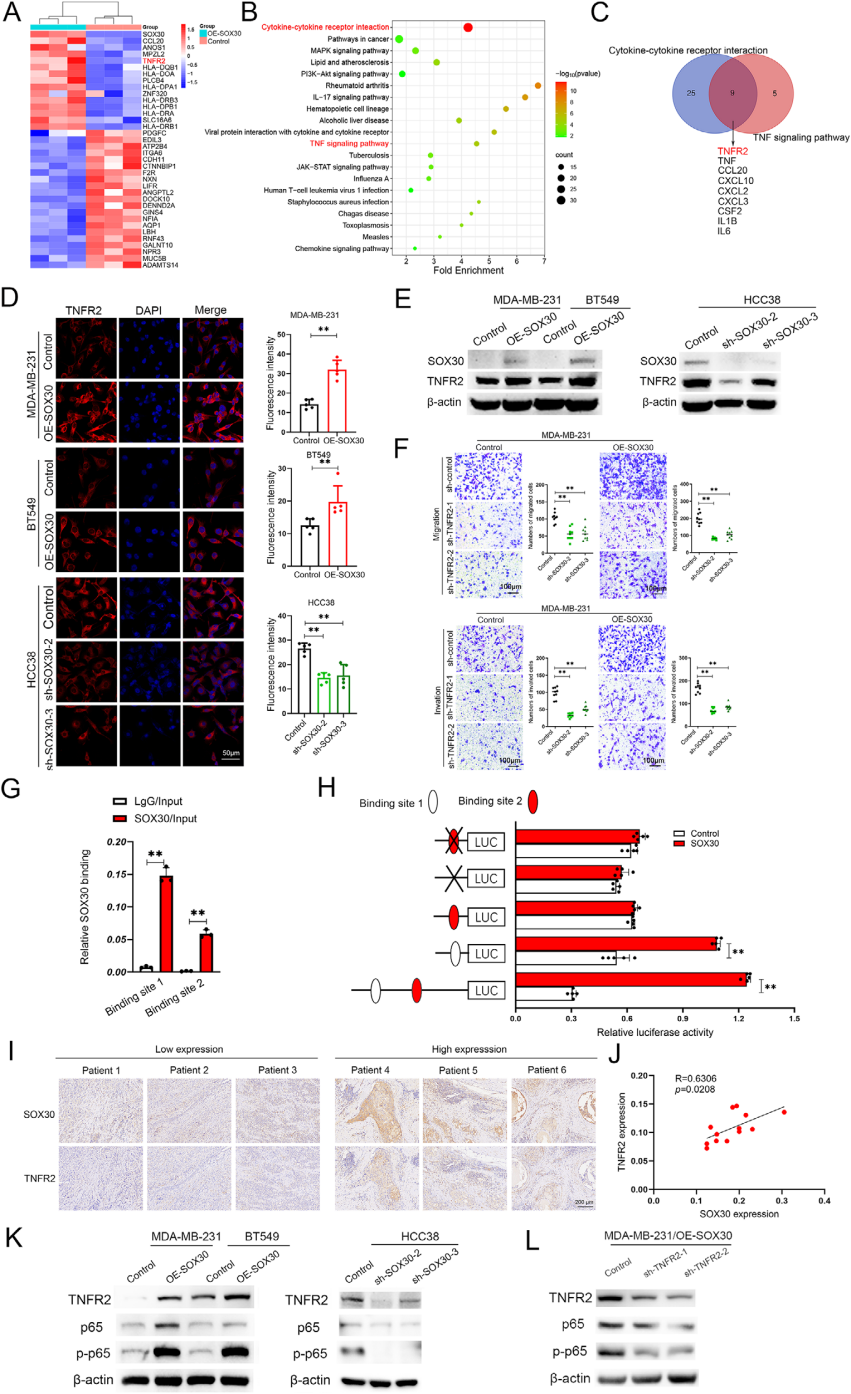
SOX30 drives TNBC metastasis via TNFR2‐mediated transcriptional activation of the NF‐κB signaling pathway. (A) Transcriptome differential gene cluster map. (B) KEGG enrichment analysis of transcriptome differential genes. (C) Venn diagram of cytokine‐cytokine receptor interaction and TNF signaling pathway. (D) Representative images of IF showed the TNFR2 expression level in MDA‐MB‐231 overexpressing SOX30 cells, BT549 overexpressing SOX30 cells, and HCC38 knockdown SOX30 cells. Bar = 50 µm. (E) Protein expression level of TNFR2 in MDA‐MB‐231 overexpressing SOX30 cells, BT549 overexpressing SOX30 cells, and HCC38 knockdown SOX30 cells detected by western blotting. (F) Effects of TNFR2 on cell migration and invasion after shRNA interference in MDA‐MB‐231 and SOX30‐overexpressing MDA‐MB‐231 cells. Bar = 100 µm. (G) The binding of SOX30 on TNFR2 promoter in MDA‐MB‐231 overexpressing SOX30 cells was tested by ChIP‐qPCR assay. (H) The effect of SOX30 on TNFR2 promoter activity was detected by a double luciferase reporter gene. (I) Representative images of IHC staining with SOX30 and TNFR2 in the SOX30‐positive and SOX30‐negative tumor tissues in TNBC patients. Bar = 200 µm. (J) The correlation between SOX30 and TNFR2 expression in TNBC patients. (K) Protein expression levels of SOX30, TNFR2, p65, and p‐p65 in MDA‐MB‐231 overexpressing SOX30 cells, BT549 overexpressing SOX30 cells, and HCC38 knockdown SOX30 cells detected by western blotting. (L) Protein expression levels of TNFR2, p65, and p‐p65 in MDA‐MB‐231 overexpressing SOX30 cells after interfering with TNFR2. ^**^: *p* < 0.01.

To investigate the molecular mechanisms underlying SOX30‐driven enhancement of migration and invasion in TNBC, we performed transcriptome sequencing on MDA‐MB‐231 cells with SOX30 overexpression and their corresponding control cells. Cluster analysis of differentially expressed genes revealed distinct transcriptional profiles between the two groups (Figure 3A). KEGG pathway enrichment analysis identified several significantly enriched signaling pathways, including cytokine‐cytokine receptor interaction, pathways in cancer, and the TNF signaling pathway (Figure 3B). Notably, the cytokine‐cytokine receptor interaction and TNF signaling pathways shared nine overlapping genes: *TNFR2*, *TNF*, *CCL20*, *CXCL10*, *CXCL2*, *CXCL3*, *CSF2*, *IL1B*, and *IL6* (Figure 3C). Among these, eight genes (excluding *TNFR2*) are well‐established downstream effectors of the NF‐κB signaling cascade.

Bioinformatics analysis using GEPIA demonstrated a strong positive correlation between SOX30 and TNFR2 expression across breast cancer samples (Figure S3A). Further analysis of the CCLE database revealed that TNFR2 expression was higher in TNBC cell lines than in other BRCA subtypes (Figure S3B). In addition, we analyzed RNA‐seq expression data from 945 primary breast cancer samples in the TCGA PanCancer Atlas, stratified by PAM50 molecular subtype. TNFR2 is significantly overexpressed in Basal‐like tumors, which are characterized by a basal‐like molecular phenotype and represent the majority of TNBC, compared to all other subtypes (Figure S3C). To validate the regulatory relationship between SOX30 and TNFR2, we performed qRT‐PCR, immunofluorescence (IF), and western blotting analyses in multiple breast cancer cell models. Notably, TNFR2 expression was significantly upregulated in SOX30‐overexpressing MDA‐MB‐231 and BT549 cells compared with control cells, while it was markedly reduced in SOX30‐knockdown HCC38 cells (Figures 3D,E and S3D,E). These findings support a role for SOX30 as a transcriptional regulator of TNFR2.

To further investigate the transcriptional regulation of TNFR2 by SOX30 in TNBC, we performed TNFR2 knockdown using shRNA in SOX30‐overexpressing MDA‐MB‐231 cells. Transwell assays demonstrated markedly reduced migration and invasion capacities in TNFR2‐depleted cells (Figure 3F), suggesting that SOX30 promotes TNBC metastasis through TNFR2. To delineate the underlying transcriptional mechanism, we conducted bioinformatic analysis using LASAGNA‐Search 2.0, which predicted two potential SOX30 binding sites within the TNFR2 (TNFRSF1B) promoter region (Figure S3F). Chromatin immunoprecipitation followed by quantitative PCR (ChIP‐qPCR) experiments provided direct evidence of SOX30 binding to the TNFR2 promoter (Figures 3G and S4A). In addition, immunofluorescence co‐localization assays showed that SOX30 and TNFR2 co‐localize in lung tissue sections from TNBC patients with lung metastasis (Figure S4B). Dual‐luciferase reporter assays further validated these findings, showing significant enhancement of both full‐length TNFR2 promoter activity and binding site 1‐specific transcriptional activity (Figure 3H). Consistently, in mouse models, TNFR2 expression was higher in SOX30‐overexpressing tumors and lower in SOX30‐knockdown tumors (Figure S4C,D). Moreover, breast cancer patients with high SOX30 expression exhibited elevated TNFR2 expression levels, whereas those with low SOX30 expression showed reduced TNFR2 expression (Figure 3I,J).

To further elucidate how SOX30 contributes to TNBC metastasis, we examined the activation status of the NF‐κB pathway by measuring total and phosphorylated p65 (p‐p65) protein levels via western blotting. In SOX30‐overexpressing MDA‐MB‐231 and BT549 cells, p65 and p‑p65 were upregulated, whereas they were downregulated in SOX30‐depleted HCC38 cells. Notably, TNFR2 knockdown reversed this upregulation of p65 and p‑p65 in SOX30 overexpressing MDA‐MB‐231 cells (Figures 3K,L and S5A,B). Immunofluorescence confirmed that SOX30 overexpression promoted nuclear accumulation of p65 (Figure S6A), and increased p65 transcriptional activity in MDA‐MB‐231 and BT549 cells, with the opposite effect observed upon SOX30 knockdown in HCC38 cells (Figure S6B). In contrast, SOX30 overexpression failed to activate p65 transcriptional activity (Figure S6C) or elevate TNFR2 protein levels (Figure S6D) in MCF7 and ZR‐75‐1 cells, implying that the TNFR2–NF‑κB axis is not a critical regulatory node in these lines. Functionally, treatment with an NF‑κB inhibitor significantly attenuated the migration and invasion of SOX30‐overexpressing MDA‐MB‐231 cells relative to controls (Figure S6E). These results suggest that SOX30 transcriptionally activates TNFR2, thereby triggering the downstream NF‑κB cascade to promote metastatic progression in TNBC.

### 2.4 SOX30 Contributes to TNBC Progression Through Reprogramming the Immunosuppressive Tumor Microenvironment

**FIGURE 4:**
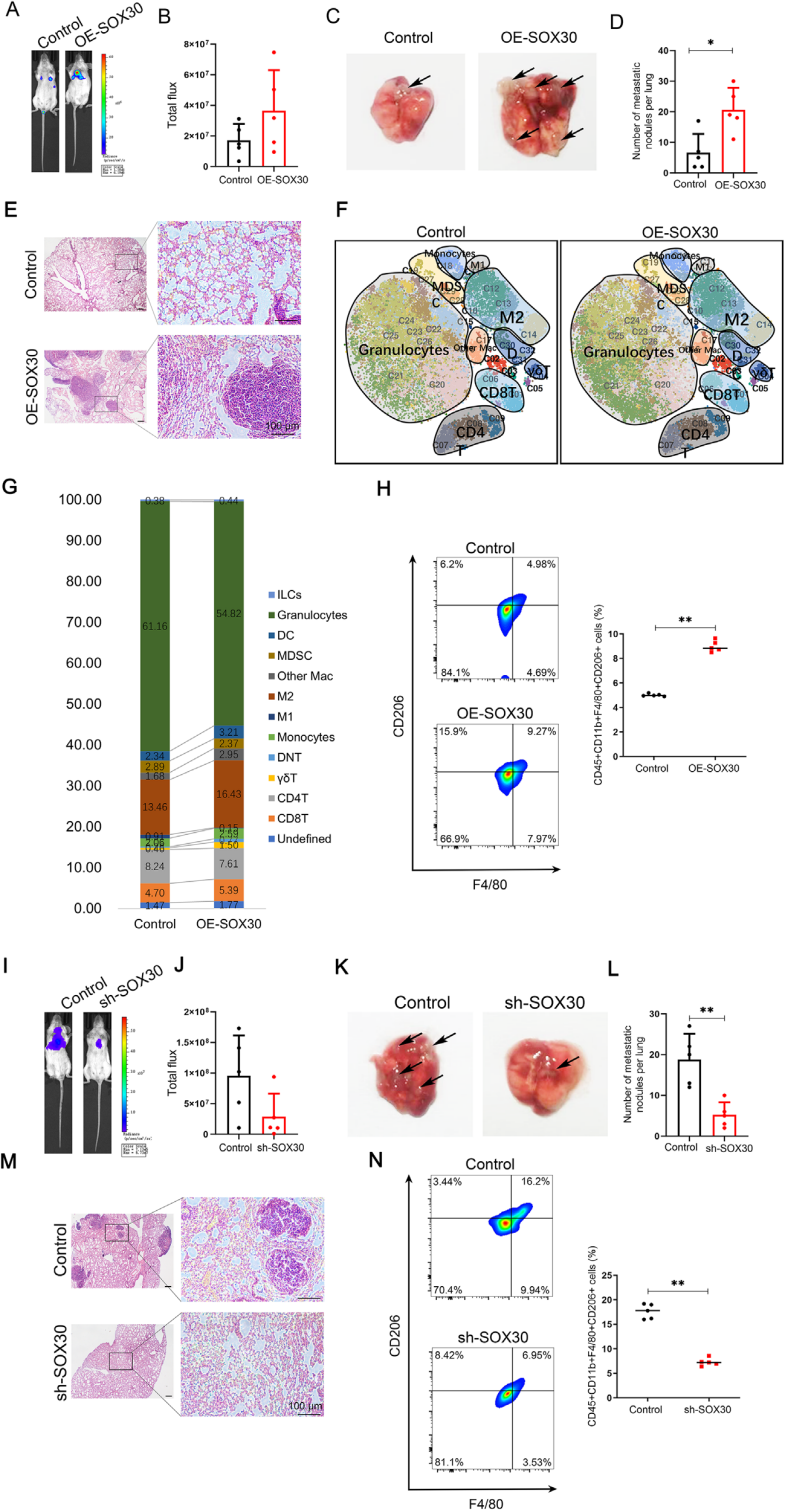
SOX30 affects the immune microenvironment. (A) The representative bioluminescent images of Balb/c mice inoculated with 4T1 cells and 4T1 overexpressing SOX30 cells. (B) Bioluminescence intensity of lung metastasis in the SOX30‐overexpressing group and control group. (C) Images of lung metastasis in the SOX30‐overexpressing group and control group. (D) Statistical graphs of nodules in the SOX30‐overexpressing group and control group. (E) H&E staining of lung tissue in the SOX30‐overexpressing group and control group. Bar = 100 µm. (F) t‐SNE plots color‐coded expression of marker for main immune cell types, based on CyTOF analysis of lung tissue from the SOX30‐overexpressing group and control group. (G) Frequency diagram of immune cell subsets in the SOX30‐overexpressing group and control group. (H) Proportion of TAMs analyzed by flow cytometry in the SOX30‐overexpressing group and control group. (I)The representative bioluminescent images of Balb/c mice inoculated with 4T1 knockdown SOX30 cells and control cells. (J) Bioluminescence intensity of lung metastasis in the SOX30‐knockdown group and control group. (K) Images of lung metastasis in the SOX30‐knockdown group and control group. (L) Statistical graphs of nodules in the SOX30‐knockdown group and control group. (M) H&E staining of lung tissue in the SOX30‐knockdown group and control group. Bar = 100 µm. (N) Proportion of TAMs analyzed by flow cytometry in the SOX30‐knockdown group and control group. ^*^: *p* < 0.05. ^**^: *p* < 0.01.

Transcriptomic analysis revealed that SOX30 predominantly influences the cytokine‐cytokine receptor interaction pathway, suggesting a potential role for SOX30 in regulating the tumor immune microenvironment. To further elucidate SOX30's regulatory role in the tumor immune microenvironment, we subsequently conducted in vivo experiments. Three weeks following tail vein injection of SOX30‐overexpressing 4T1 cells or control cells, the SOX30‐overexpression group exhibited significantly enhanced lung metastatic capacity compared to controls (Figure 4A–E). CyTOF analysis of pulmonary tissues revealed that M2‐polarized TAMs were enriched higher in the SOX30‐overexpression group than the control group. Conversely, antitumor immune components, such as M1‐polarized macrophages and granulocytes, were significantly depleted in the SOX30‐overexpression group (Figures 4F,G and S7). Given that TAMs are a major component of the breast cancer microenvironment and primarily exert pro‐tumor effects, we propose that SOX30 may promote the malignant progression of TNBC predominantly through TAM recruitment and polarization. Furthermore, supernatant from SOX30‐overexpressing cultures promoted M2 polarization of macrophages, while supernatant from SOX30‐knockdown cultures inhibited M2 polarization (Figure S8A). However, SOX30 had no significant effect on M1 polarization (Figure S8B). To further assess the effect of SOX30 on M2 macrophage polarization, we used CD163 to assess the M2 polarization state in co‐culture models. The results indicated that SOX30‐overexpressing cells promoted M2 polarization of macrophages, while SOX30‐knockdown cells inhibited M2 polarization (Figure S8C). Flow cytometry validation demonstrated that TAMs were increased in the SOX30‐overexpression group (Figure 4H). Consistently, SOX30 knockdown significantly inhibited lung metastasis in murine models, accompanied by a significant attenuation of M2‐polarized macrophage infiltration within lung tissues. (Figure 4I–N). These findings demonstrate that SOX30 plays a key role in reprogramming the immunosuppressive tumor microenvironment by promoting TAM recruitment and polarization. This immune modulation significantly contributes to the metastatic progression of TNBC.

### 2.5 SOX30 Promotes Metastatic Progression via TNFR2–NF‐κB–CCL20/CCR6 Axis‐Mediated Recruitment of TAMs

**FIGURE 5:**
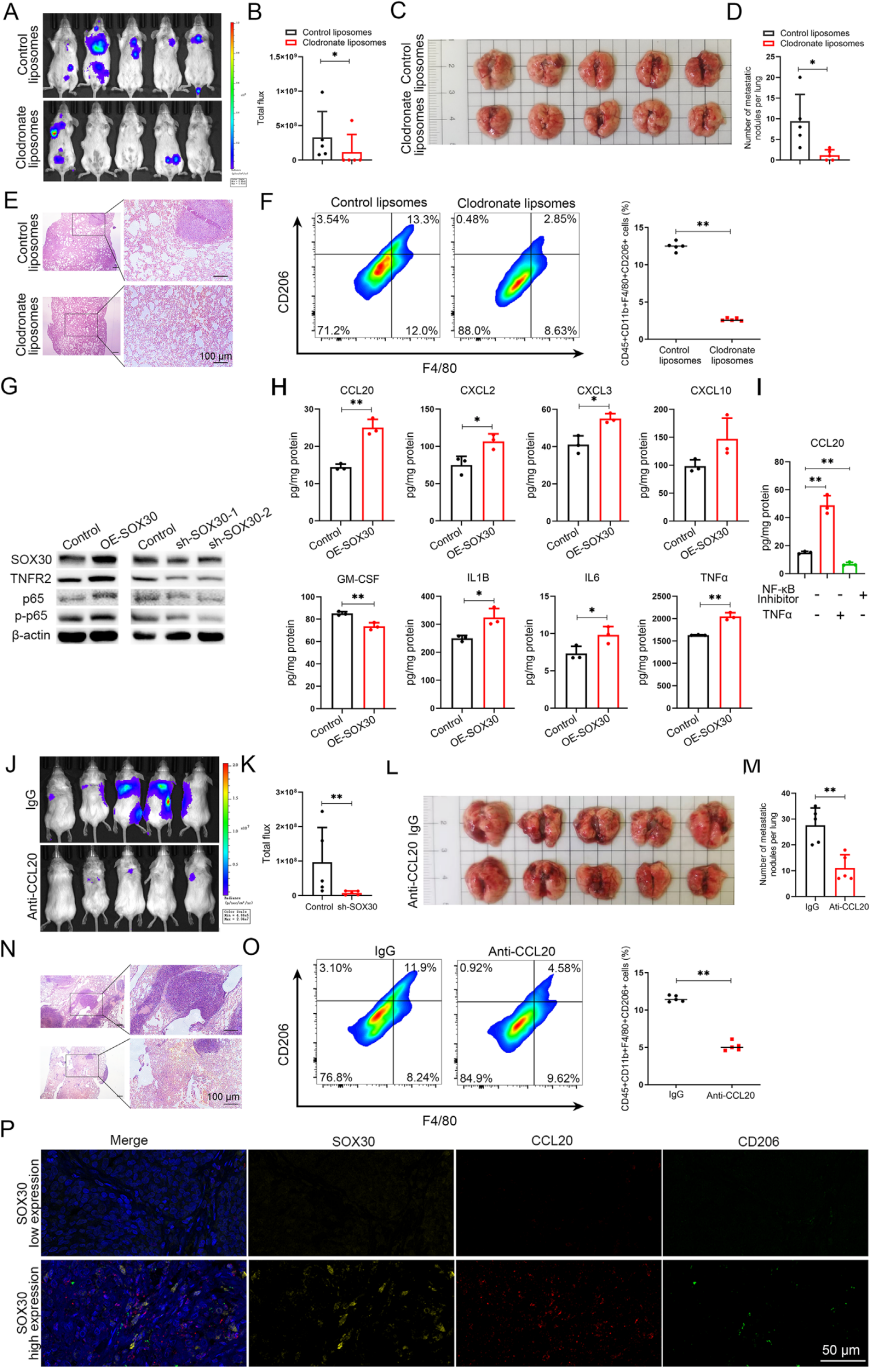
SOX30 promotes metastatic progression via TNFR2–NF‐κB–CCL20/CCR6 axis‐mediated recruitment of TAMs. (A) The representative bioluminescent images of Balb/c mice inoculated with 4T1 overexpressing SOX30 cells were treated with control liposomes or clodronate liposomes. (B) Bioluminescence intensity of lung metastasis in the control and clodronate liposomes groups. (C) Images of lung metastasis in the control and clodronate liposomes groups. (D) Statistical graphs of nodules in the control and clodronate liposomes groups. (E) H&E staining of lung tissue in the control and clodronate liposomes groups. Bar = 100 µm. (F) Proportion of TAMs analyzed by flow cytometry. (G) Protein expression level of SOX30, TNFR2, p65, p‐p65 in 4T1 overexpressing SOX30 cells and 4T1 knockdown SOX30 cells detected by western blotting. (H) Elisa analysis of CCL20, CXCL2, CXCL3, CXCL10, GM‐CSF, IL1B, IL6 and TNF‐α in 4T1 overexpressing SOX30 cells and control cells. (I) The effect of NF‐κB activator and inhibitor to CCL20 by elisa analysis. (J) The representative bioluminescent images of Balb/c mice inoculated with 4T1 overexpressing SOX30 cells were treated with control IgG or anti‐CCL20 antibody. (K) Bioluminescence intensity of lung metastasis in the control and anti‐CCL20 antibody groups. (L) Images of lung metastasis in the control and anti‐CCL20 antibody groups. (M) Statistical graphs of nodules in the control and anti‐CCL20 antibody groups. (N) H&E staining of lung tissue in the control and anti‐CCL20 antibody groups. Bar = 100 µm. (O) Proportion of TAMs analyzed by flow cytometry in the control and anti‐CCL20 antibody groups. (P) Multiplex immunofluorescence assay of SOX30, CCL20, and CD206 in the tissue samples of lung metastasis of patients with TNBC. Bar = 50 µm. ^*^: *p* < 0.05. ^**^: *p* < 0.01.

To investigate the mechanistic role of TAMs in SOX30‐driven TNBC metastasis, we depleted TAMs using clodronate liposomes. This depletion substantially attenuated pulmonary metastatic progression (Figure 5A–F). Furthermore, we performed rescue experiments using TAMs educated by SOX30‐overexpressing 4T1 cells compared with those educated by control cells. Our results demonstrated that the TAMs educated by SOX30‐overexpressing TNBC cells significantly promoted lung metastasis (Figure S9A–E), further supporting the essential role of TAMs in SOX30‐driven metastasis.

To elucidate how SOX30 facilitates TAM recruitment, we examined the TNFR2–NF‐κB pathway in 4T1 cells. Western blot analysis of 4T1 cells with SOX30 overexpression or knockdown revealed that protein levels of TNFR2, p65, and p‐p65 were upregulated in SOX30‐overexpressing cells and downregulated in SOX30‐knockdown cells (Figures 5G and S10A). To determine whether SOX30 promotes metastasis through an intracellular signaling pathway, we evaluated its effects on cell migration and invasion in 4T1 cells. Overexpression of SOX30 significantly enhanced the migration and invasion of 4T1 cells, while SOX30 knockdown conversely suppressed these malignant phenotypes (Figure S10B–E). To further investigate the underlying mechanism, we performed ELISA analysis, which revealed that most downstream cytokines of the NF‐κB pathway were elevated, with CCL20 showing the most pronounced increase (Figure 5H). Furthermore, activation of NF‐κB upregulated CCL20 expression, whereas pharmacological inhibition of NF‐κB signaling significantly suppressed CCL20 production (Figure 5I). Multiplex immunofluorescence staining of TNBC lung metastasis tissue showed that CCR6+ macrophages were enriched in the tumor stroma and often located adjacent to CK+ tumor nests (Figure S11), suggesting a potential paracrine interaction between CCL20‑producing tumor cells and CCR6+ macrophages. In addition, in vitro macrophage chemotaxis assays demonstrated that CCL20 significantly promoted M2 macrophage migration (Figure S12). In vivo neutralization of CCL20 using a monoclonal antibody treatment significantly attenuated pulmonary metastatic progression and reduced TAM infiltration (Figure 5J–O). Moreover, multiplex immunofluorescence staining revealed that pulmonary metastatic tissue from TNBC patients with high SOX30 expression exhibited elevated levels of CCL20 and CD206 (Figure 5P). IHC analysis showed that the SOX30‐high expression group exhibited significantly higher levels of TNFR2, CCL20, and M2 macrophage markers (CD163, CD206) compared to the SOX30‐low expression group (Figure S13). These results revealed that SOX30 may orchestrate TAM infiltration via the TNFR2–NF‐κB–CCL20/CCR6 axis.

### 2.6 Combination Therapy Targeting CCR6 and TNFR2 Synergistically Suppresses SOX30‐Mediated TNBC Metastasis

**FIGURE 6:**
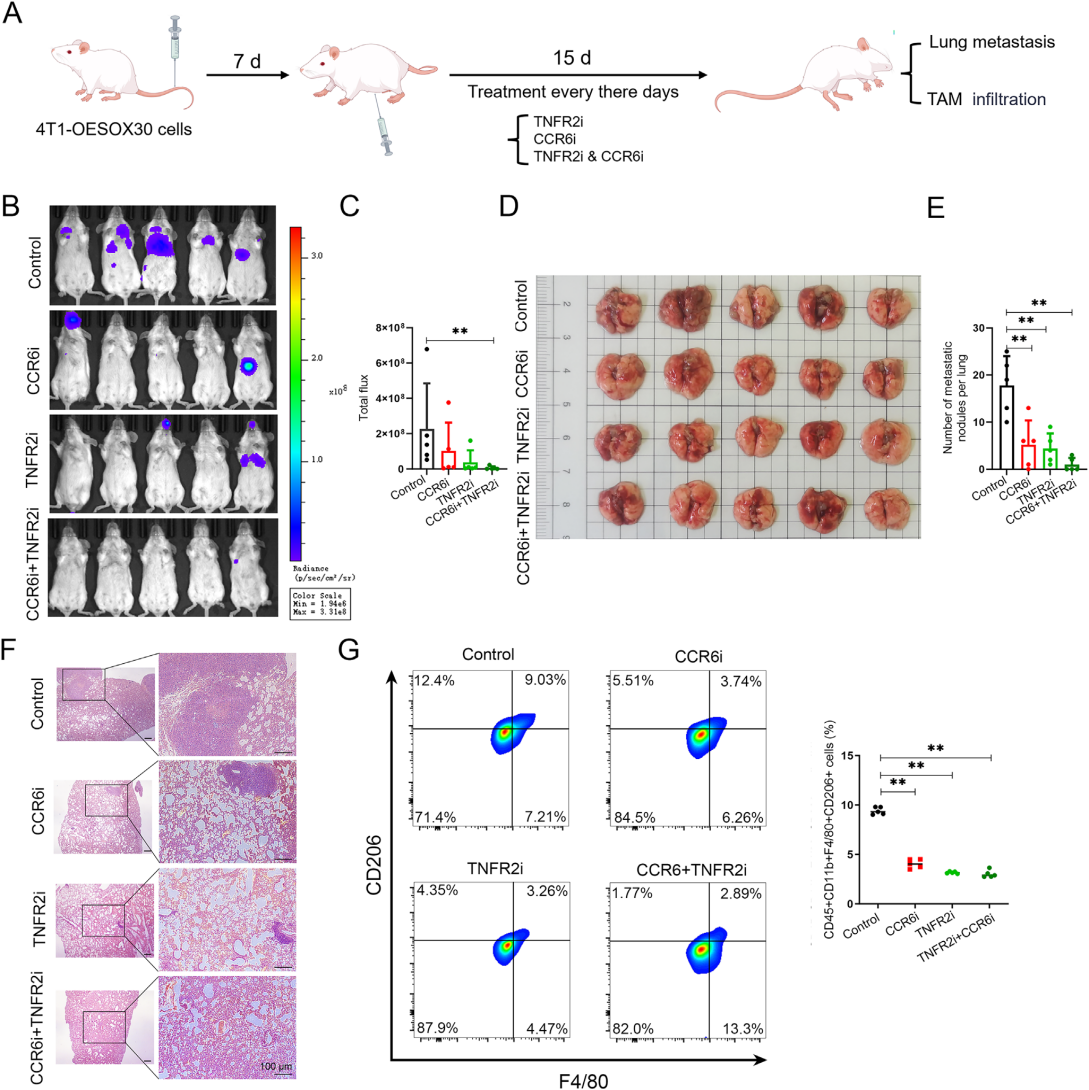
Combination treatment of CCR6 inhibitor with TNFR2 inhibitor dramatically inhibits SOX30‐mediated TNBC metastasis. (A) Schematic depicting the experimental setup. (B) Representative bioluminescent images of Balb/c mice inoculated with 4T1 cells overexpressing SOX30 and further treated with CCR6 inhibitor, TNFR2 inhibitor etanercept, and combined therapy with CCR6 inhibitor and etanercept. (C) Bioluminescence intensity of lung metastasis in the CCR6 inhibitor, TNFR2 inhibitor etanercept, combined therapy with CCR6 inhibitor and etanercept, and control groups. (D) Images of lung metastasis in the CCR6 inhibitor, TNFR2 inhibitor etanercept, combined therapy with CCR6 inhibitor and etanercept, and control groups. (E) Statistical graphs of nodules in the CCR6 inhibitor, TNFR2 inhibitor etanercept, combined therapy with CCR6 inhibitor and etanercept, and control groups. (F) H&E staining of lung tissue in the CCR6 inhibitor, TNFR2 inhibitor etanercept, combined therapy with CCR6 inhibitor and etanercept, and control groups. Bar = 100 ‌µm. (G) Proportion of TAMs analyzed by flow cytometry in the CCR6 inhibitor, TNFR2 inhibitor etanercept, combined therapy with CCR6 inhibitor and etanercept, and control groups. ^**^: *p* < 0.01.

Given the pivotal role of the SOX30–TNFR2–NF‐κB–CCL20/CCR6 axis in remodeling the tumor immune microenvironment and promoting TNBC metastasis, we investigated the therapeutic potential of combining a CCR6 inhibitor with TNFR2 blockade (Figure 6A). We treated animal models with a selective TNFR2 antagonist (anti‐TNFR2 neutralizing antibody). Pharmacological inhibition of TNFR2 signaling significantly attenuated metastatic progression (Figure S14A–E). Moreover, both TNFR2 inhibitor (Etanercept) and CCR6 inhibitor monotherapies exhibited substantial anti‐metastatic activity compared to control groups (Figure 6B–F). Strikingly, combination therapy demonstrated synergistic suppression of lung metastasis compared to single‐agent interventions, suggesting enhanced therapeutic potential through dual pathway inhibition (Figure 6B–F). Moreover, therapeutic intervention with either a CCR6 inhibitor, TNFR2 blockade, or their combination significantly reduced TAM infiltration (Figure 6G).

## 3 Discussion

Metastasis remains the leading cause of mortality in TNBC patients [4]. However, the underlying molecular mechanisms remains incompletely understood. Through an integrated approach combining cellular studies, animal models, bioinformatics analyses, and clinical sample validation, we identified SOX30 as a novel metastasis‐specific regulator in TNBC. Importantly, the bidirectional modulation of SOX30 on both tumor cell behavior and the immune microenvironment support its potential as a therapeutic target.

Tumor necrosis factor‐alpha (TNF‐α) exerts its biological effects through two distinct receptors. Among these, TNFR2 is predominantly expressed on tumor cells and immunosuppressive immune cells, where it mediates TNF signaling [20]. Unlike TNFR1, which contains a death domain, TNFR2 lacks this critical structural motif and instead exhibits tumor‐promoting properties [21]. For example, Tanimura et al. demonstrated that TNFR2‐mediated upregulation of matrix metalloproteinase‐9 (MMP‐9) secretion critically enhances the invasive capacity of cholangiocarcinoma cells [22]. In breast cancer, TNFR2 expression is positively correlated with tumor size, advanced clinical stage, and higher pathological grade [23]. Importantly, preclinical studies using anti‐TNFR2 monoclonal antibodies have demonstrated significant growth‐inhibitory effects on breast cancer cells in both in vitro models and xenograft systems [24]. Contributing to these accumulating evidences, our study demonstrates that SOX30 drives TNBC metastasis by transcriptionally activating TNFR2 expression. NF‐κB is a key downstream effector of TNF‐α signaling. In addition to TNFR1, emerging evidence demonstrates that TNFR2 can also activate the NF‐κB pathway. For instance, N‐acetylglucosaminyltransferase III attenuates chemoresistance through suppression of P‐glycoprotein expression via the TNFR2–NF‐κB axis [25], and TNFR2 promotes cell proliferation, migration, and invasion in pancreatic cancer through NF‐κB activation [26]. Given that numerous NF‐κB downstream targets were significantly upregulated in the SOX30 overexpression group, our findings further suggest that SOX30 may enhance TNBC metastasis through TNFR2‐mediated activation of the NF‐κB signaling pathway. Given the critical role of this axis in tumor progression, targeting SOX30 or its downstream effectors may represent a promising therapeutic strategy.

While our data point to NF‐κB as a key downstream effector of the SOX30–TNFR2 axis, we acknowledge that NF‐κB does not function in isolation. Parallel signaling pathways such as MAPK and AKT are known to engage in extensive crosstalk with NF‐κB under various cellular stress conditions and may collectively regulate downstream responses. Although our current data do not experimentally exclude the contributions of these pathways, our data suggest that NF‐κB signaling plays a predominant role in the observed effects. Future studies are warranted to fully delineate this regulatory network. In particular, combinatorial use of specific inhibitors or multi‐pathway gene knockout models will help clarify the hierarchical relationship and synergistic/antagonistic interactions between NF‐κB and MAPK/AKT in a specific cellular context. In summary, this work establishes a central role for NF‐κB in TNBC metastasis, while also providing a clear rationale for future investigations aimed at comprehensively mapping the signal integration mechanisms within this regulatory network.

The role of SOX30 has been studied in several cancers, including lung, ovarian, prostate, and bladder carcinomas, where it primarily exhibits tumor‐suppressive functions, including inhibiting cancer cell proliferation and metastasis [11, 12, 13, 14, 15, 27]. Existing studies have demonstrated SOX30‐mediated inhibition of Wnt/β‐catenin signaling as a key mechanism underlying its anti‐metastatic effects in both lung and prostate cancers [13, 27]. Contrary to these findings, our experimental models revealed a pro‐metastatic function of SOX30 in TNBC via TNFR2–NF‐κB signaling and tumor microenvironment remodeling. This functional discrepancy may arise from the tumor heterogeneity of SOX30 in different cancer types or subtypes. SOX30 expression is significantly elevated in TNBC compared to other breast cancer subtypes, which may be driven by subtype‐specific epigenetic alterations such as promoter hypomethylation or enhancer activation that are enriched in TNBC. In contrast, in cancers where SOX30 acts as a tumor suppressor, its expression is often silenced via promoter hypermethylation [11], suggesting that the functional outcome of SOX30 may depend on its expression level and the accompanying epigenetic landscape. Furthermore, the SOX30‐TNFR2 axis is likely TNBC‐specific. Both SOX30 and TNFR2 are expressed at higher levels in TNBC than in other subtypes. In non‐TNBC cells, overexpression of SOX30 neither increases the expression level of TNFR2 nor activates the p65 activity. This suggests that SOX30 functions in a context‐dependent manner. For example, SOX30 has been reported to directly bind to and activate p53 in lung cancer [11]. Given that TNBC frequently harbors p53 mutations, this tumor‐suppressive arm is likely disabled, shifting the balance toward its oncogenic functions. Moreover, the TNBC microenvironment is characterized by high levels of inflammation, immune infiltration, and NF‐κB activation. Our finding that SOX30 upregulates CCL20, a chemokine that recruits M2 macrophages, suggests that SOX30 may promote tumor progression not only through cell‐autonomous mechanisms but also by remodeling the tumor immune microenvironment. This mechanism may be less relevant in cancer types where SOX30 is known to act as a tumor suppressor. Future studies should systematically delineate the subtype‐specific molecular mechanisms underlying SOX30's functional pleiotropy across breast cancer subtypes.

This functional dichotomy, however, is not without precedent—either for SOX30 itself or for transcription factors and signaling molecules in cancer biology more broadly. Critically, previous work has already demonstrated that SOX30 exhibits subtype‐specific functions even within a single organ. In non‐small cell lung cancer, SOX30 suppresses metastasis in lung adenocarcinoma (ADC) through direct binding to the CTNNB1 (β‐catenin) promoter and inhibition of Wnt/β‐catenin signaling, whereas it has no effect on metastasis in lung squamous cell carcinoma (SSC) because the β‐catenin promoter is inaccessible to SOX30 in SCC cells [12, 13]. Notably, that study reported that SOX30 is a favorable and independent prognostic factor in ADC patients, yet an unfavorable and independent prognostic factor in SSC patients. This within‐organ functional divergence establishes the principle that SOX30's transcriptional output—and consequently its role in tumor progression—is governed by subtype‐specific chromatin landscapes and cofactor availability rather than by any intrinsic, invariant property of the protein.

The functional switch of transcription factors and signaling molecules between oncogenic and tumor‐suppressive roles is not unique to SOX30. The NOTCH1 case is particularly instructive: Lobry et al. [28] articulated how the functional output of NOTCH1 is ultimately determined by the cellular context‐the chromatin landscape, available cofactors, and cell‐type‐specific signaling milieu. Lim et al. [29] subsequently demonstrated that NOTCH1 functions as a tumor suppressor in small‐cell lung cancer (SCLC) while simultaneously acting as an oncogene in other contexts, with the dichotomy governed by differential target gene activation. Our findings with SOX30 align with this emerging paradigm ‐extending it to a member of the SOX transcription factor family. As the field increasingly recognizes, “context is everything” in transcriptional regulation [30].

Several non‐mutually exclusive mechanisms may account for this context‐dependent reprogramming. First, SOX family transcription factors are well established to require cooperative interactions with tissue‐ and cell‐type‐specific partner factors (e.g., POU, PAX, and homeodomain proteins) for high‐affinity, sequence‐specific DNA binding [31, 32]. The repertoire of available SOX30 partner factors in TNBC likely differs substantially from that in lung or ovarian epithelial cells, potentially redirecting SOX30 to the TNFR2 promoter. Second, TNBC is characterized by constitutively elevated basal NF‐κB activity driven by chronic inflammatory signaling and loss of negative regulators [33], which may create a permissive chromatin environment at NF‐κB pathway gene promoters, including TNFR2.

Nevertheless, the mechanistic basis of the SOX30 functional switch between its tumor‐suppressive and pro‐metastatic roles warrants further investigation. While our data suggest that differential target gene selection underlies this switch, the molecular determinants of this context‐dependent reprogramming remain to be fully elucidated. Systematic comparative ChIP‐seq and ATAC‐seq analyses of SOX30 binding and chromatin accessibility across TNBC and non‐TNBC breast cancer cell lines, as well as identification of SOX30‐interacting partner proteins and post‐translational modifications by IP‐mass spectrometry, are important directions for future research to define the precise mechanisms governing SOX30 target gene selectivity.″

Accumulating evidence indicates that TAMs promote cancer metastasis [18]. For example, exosomal miR‐934 drives liver metastasis in colorectal cancer by inducing M2 macrophage polarization through tumor‐derived secretions [17], and inhibition of SLITRK4 suppresses colorectal cancer proliferation and hepatic metastasis by dually modulating the PI3K/AKT/NF‐κB signaling axis and TAM activity [34]. Our study demonstrates that, in addition to activating the SOX30/TNFR2/NF‐κB signaling pathway, SOX30 promotes metastatic progression by mediating the recruitment of TAMs to metastatic niches. The chemokine CCL20 has been mechanistically implicated in macrophage recruitment through engagement of the CCR6 receptor, a conserved pathway that has been corroborated across multiple malignancies. In glioma models, FDPS was demonstrated to promote tumor progression and TAM infiltration by modulating CCL20 through the Wnt/β‐catenin signaling pathway [35]. In esophageal squamous cell carcinoma, FOXO1 has been reported to facilitate M2 macrophage infiltration via coordinated secretion of CCL20 and CSF‐1 [36]. Moreover, extensive experimental evidence has established CCL20 as a downstream effector of NF‐κB signaling [37, 38, 39]. In our study, we uncovered a positive feedback regulatory axis, the SOX30–TNFR2–NF‐κB–CCL20/CCR6 axis, that mediates crosstalk between TNBC cells and TAMs. Our results showed that NF‐κB inhibition completely abolished the SOX30‐induced enhancement of cell migration and invasion. Since CCL20 is a downstream effector of the NF‐κB pathway, these findings suggest that the entire SOX30–TNFR2–NF‐κB axis, including CCL20, is necessary for the observed phenotype. We acknowledge, however, that these experiments do not directly dissect the specific contribution of CCL20 to TNBC cell migration versus its role in macrophage recruitment. Direct gain‐ and loss‐of‐function studies of CCL20 in TNBC cells would be an important avenue for future investigation. In addition, the functional regulation of TAMs by SOX30 and its underlying mechanisms remain unexplored. Future investigations are required to delineate its immunomodulatory roles on TAMs and effector mechanisms specifically within metastatic niche contexts.

Substantiated evidence suggests that therapeutic targeting of TAMs represents a promising strategy for the development of novel cancer immunotherapies [40]. Combination immunotherapy is increasingly regarded as an effective approach to overcome the limitations of single‐agent immune checkpoint blockade therapy [41]. Etanercept, a TNFR2 inhibitor, is a recombinant fusion protein consisting of TNFR2 and the Fc portion of human IgG1, used to treat autoimmune diseases by blocking TNF activity to reduce inflammatory responses [42]. Emerging evidence from recent studies has demonstrated that etanercept exhibits promising therapeutic potential in cancer treatment. In vitro experiments demonstrated that etanercept significantly suppressed breast cancer cell viability, induced cell apoptosis and cell cycle arrest, concomitant with marked inhibition of NF‐κB signaling pathway activation [43]. A phase II study of etanercept in metastatic breast cancer patients demonstrated its safety and biological activity in this population [44]. In pancreatic cancer, etanercept treatment in mouse models improved chemosensitivity [45]. In our investigation, monotherapeutic administration of a CCL20‐neutralizing antibody, a selective CCR6 inhibitor, or etanercept demonstrated significant anti‐metastatic effects in breast cancer models. Notably, the combination of CCR6 inhibition and TNFR2 blockade exhibited significantly greater suppression of TNBC metastasis compared to monotherapy. Several factors may contribute to the observed phenomenon. Upon inhibition of the upstream component, residual activated signals may still undergo substantial amplification via downstream signaling pathways, or the downstream pathway may be activated through other bypass mechanisms. In general, our findings suggest that targeting TNFR2 in conjunction with CCL20–CCR6 blockade may offer a promising therapeutic approach aimed at overcoming the challenge of TNBC metastasis, circumventing the limitations of current immune checkpoint‐based regimens. Subsequent studies warrant systematic exploration of SOX30‐targeted therapeutic interventions, particularly leveraging its master regulatory role in tumor microenvironment reprogramming.

**FIGURE 7:**
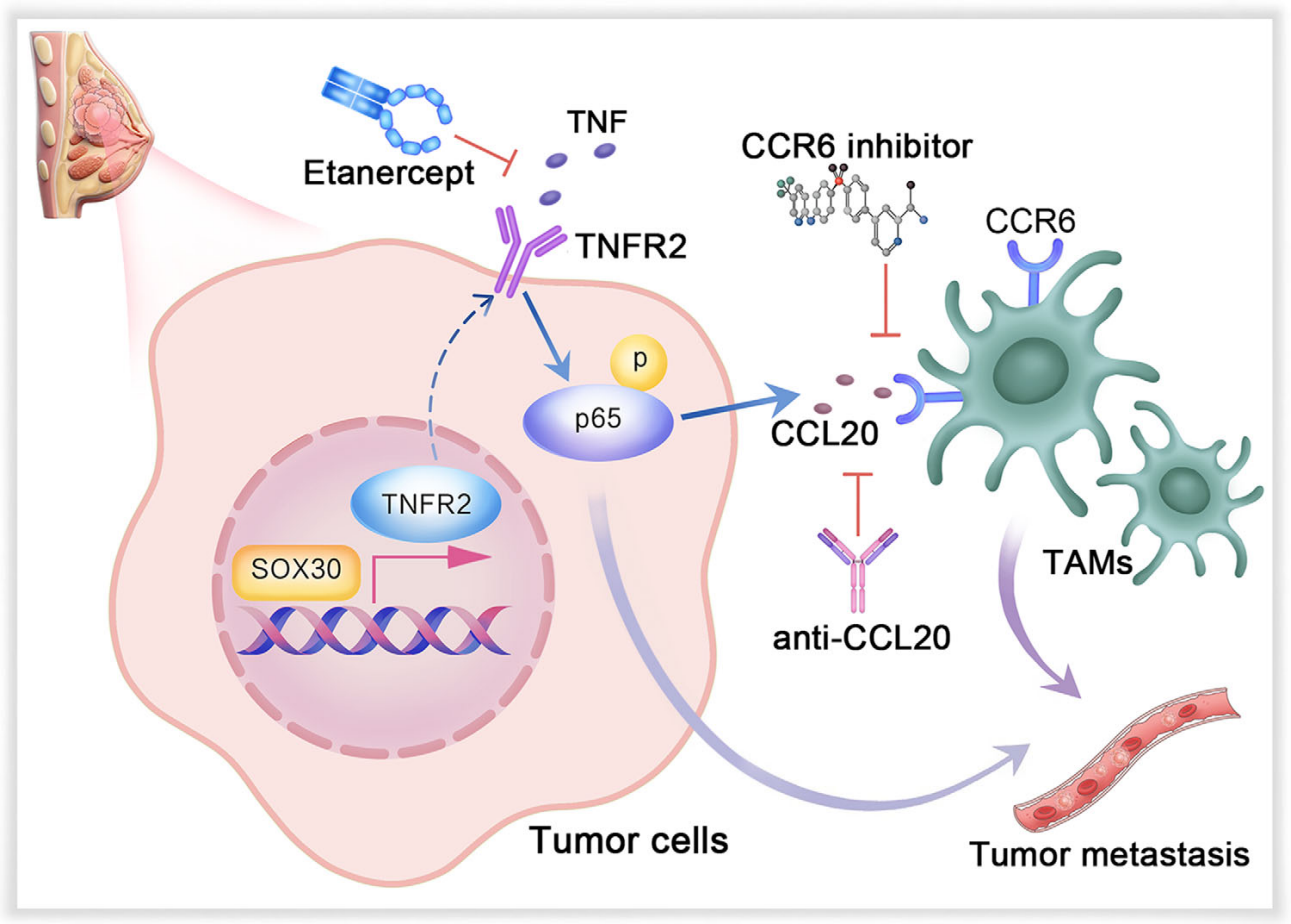
Schematic illustration of the regulatory mechanism by which SOX30 promotes breast cancer metastasis.

In conclusion, our study defines a novel SOX30–TNFR2–NF‐κB signaling axis that intricately orchestrates both cell‐autonomous metastatic programming and non‐cell‐autonomous immune microenvironment remodeling (Figure 7). Therapeutically, dual blockade of TNFR2 and the CCL20/CCR6 axis exhibits synergistic efficacy against metastatic TNBC. Our findings systematically delineate the regulation network governing TNBC metastasis progression while providing a combination therapy to improve treatment efficacy in TNBC patients.

## 4 Experimental Section

### 4.1 Cell Cultures

The human breast cancer cell lines were cultured under standard conditions (37°C, 5% CO_2_) using an incubator (SANYO MCO‐175, Japan). Specifically, MCF‐7, T47D, MDA‐MB‐231, and 4T1 were cultured in high‐glucose Dulbecco's Modified Eagle's medium (DMEM, 4.5 g/L glucose, HyClone, USA) with 10% fetal bovine serum (Biochannel, Nanjing, China). SK‐BR‐3, ZR‐75‐1, HCC1937, HCC1806, HS578T, HCC38, and BT549 cells were maintained in RPMI‐1640 medium (HyClone) with 10% fetal bovine serum (Biochannel, Nanjing, China).

### 4.2 Expression and Prognosis Analysis

Tissue‐specific expression profiles were analyzed through the TCGA database and Breast Cancer Gene‐Expression Miner v4.9 (bcGExM; http://bcgenex.ico.unicancer.fr/BC‐GEM/GEM‐requete.php) [46], while cellular expression patterns in BRCA subtype cell lines were examined using the CCLE database (https://registry.opendata.aws/ccle/) [47]. Prognostic significance was subsequently evaluated using the Kaplan–Meier Plotter (https://kmplot.com/analysis/) [48].

### 4.3 RNA Isolation and qRT‐PCR

Total RNA was extracted from breast cancer cells using TRIzol reagent (Servicebio, Wuhan, China. 1.0 µg of total RNA was subjected to reverse transcription with PrimeScript RT Reagent Kit with gDNA Eraser (Takara, Dalian, China). Quantitative real‐time PCR (qRT‐PCR) was performed using TB Green Premix Ex Taq II (TaKaRa) on a CFX Connect Real‐Time PCR Detection System (Bio‐Rad, Hercules, CA, USA as previously described [12]. The following primers were used in this study. The forward primer sequences of SOX30: GATGTCCCGCTCACCGTGTTGC, the reverse primer sequences of SOX30: GACAGGGCTTGGGCTCTGGACT. TNFR2 forward primers: AAGGAGAAGGCATGAAAT, TNFR2 reverse primers: CCAGTCTGACATCTTGAT. β‐actin (forward: CCACGAAACTACCTTCAACTCC, reverse: GTGATCTCCTTCTGCATCCTGT) served as the internal control. The relative expression levels were calculated using the 2^−ΔΔct^ method.

### 4.4 Protein Extraction and Western Blotting

Western blotting analysis was performed as previously described [12]. Briefly, cell proteins were resolved using RIPA lysis buffer (Beyotime, Shanghai, China) supplemented with phenylmethanesulfonyl fluoride protease inhibitor (Beyotime). The following primary antibodies were employed in this study: SOX30 rabbit polyclonal antibody (1:1000; abcam, ab272553), TNFR2 rabbit monoclonal antibody (1:1000; abcam; ab109322). NF‐κB p65 polyclonal antibody (1:2000; proteintech; 10745‐1‐AP), phospho‐ NF‐κB p65 (S529) recombinant rabbit monoclonal antibody (1:2000; HUABIO; ET1604‐27)

### 4.5 Plasmid Construction and Cell Transfection

The human SOX30 overexpression plasmid and mouse SOX30 overexpression plasmid was synthesized and subsequently subcloned into the GV492 lentiviral vector (Ubi‐MCS‐3FLAG‐CBh‐gcGFP‐IRES‐puromycin) or GV260 lentiviral vector (Ubi‐MCS‐firefly_Luciferase‐IRES‐Puromycin), respectively. The constructed GV492‐SOX30 plasmid and GV260‐SOX30 plasmid were sequence‐verified through vector sequencing. Lentiviral vectors for SOX30 overexpression (GV492‐SOX30) and knockdown constructs (GV112‐shSOX30) were generated through co‐transfection with lentiviral packaging plasmids (pHelper 1.0 and pHelper 2.0) in HEK293T cells. Three distinct shRNA sequences targeting human SOX30 were designed: shRNA1 (CCTCTAAGTGTTTCCAATGTA), shRNA2 (GCTTGGGTTAGAGTGGAACAA), and shRNA3 (CCCTTCAGTAAGGACAGAAA), with a scrambled shRNA sequence serving as a negative control. Three distinct shRNA sequences targeting mouse SOX30 were designed: shRNA1 (CCTTTCAGAGACTATCCAGAT), shRNA2 (GCCTCCCTTTGGCTATGGAAA), and shRNA3 (GCCTTCTGAGTTGATACGGTT), with a scrambled shRNA sequence serving as a negative control. Viral particles were harvested and used to infect breast cancer cell lines (MDA‐MB‐231, BT549, HCC38, HCC1937, 4T1) using Enhanced Infection Solution (GENE‐467, Jikai, Shanghai).

For TNFR2 knockdown experiments, three targeting shRNA sequences (shRNA1: TGCCGGCTCAGAGAATACTAT; shRNA2: GCCAGACCAGGAACTGAAACA; shRNA3: AGCCTTGGGTCTACTAATAAT) and a scrambled control were cloned into pSGU6_RFP_Neo‐homo‐GAPDH plasmid. These constructs were transfected into MDA‐MB‐231 cells using ViaFect Transfection Reagent (Promega).

### 4.6 Cell Proliferation Assay

Cell proliferation was evaluated using the MTS assay as previously described [11]. MDA‐MB‐231, BT549, ZR‐75‐1, and MCF7 cells were plated at 4 × 10^3^ cells per well on 96‐well plates, respectively, and transfected with SOX30 lentivirus or vector control. For knockdown, MDA‐MB‐231‐OE^SOX30^ stable cells, HCC38, and HCC1937 were plated at the same density and transduced with SOX30‐targeting shRNA lentiviral particles or non‐targeting control shRNA. Cell proliferation was assessed at 0, 24, 48, and 72 h post‐transduction using the CellTiter 96 Aqueous One Solution Cell Proliferation Assay (Promega), following the manufacturer's recommended protocol.

### 4.7 Cell Migration and Invasion Assays

Cell migration and invasion assays were performed using 24‐well transwell chambers (8 µm pore size; Corning Incorporated, Acton, MA, USA) as previously described [12]. Briefly, treated cells were trypsinized, resuspended in serum‐free medium (DMEM or RPMI‐1640, selected based on cell type requirements), and seeded in the upper chamber at a density of 5 × 10^4^ cells/well. The lower chamber was filled with complete medium containing 10% FBS as a chemoattractant. After 16 h of incubation at 37°C with 5% CO_2_, migrated cells on the lower membrane surface were fixed with 4% paraformaldehyde, stained with 0.1% crystal violet, and quantified using an inverted microscope (five random fields per well). For invasion assays, the upper chamber of the transwell (Corning, NY, USA) was pre‐coated with Matrigel matrix (1:8 dilution in serum‐free medium; Corning) and polymerized at 37°C for 30 min before cell seeding. Subsequent procedures were performed in the same way as those in the cell migration experiment described above.

### 4.8 Digital Gene Expression (DGE) Profiling

MDA‐MB‐231‐OE^SOX30^ stable cells and control cells were cultured in DMEM medium for 72 h, harvested, and snap‐frozen in liquid nitrogen. Total RNA was extracted as described above. Sample preparation and DGE sequencing were performed by BGI/HuaDa JiYin‐Wuhan. Differentially expressed genes were classified and annotated using gene ontology (GO) and Kyoto Encyclopedia of Genes and Genomes (KEGG) pathway analysis.

### 4.9 Luciferase Reporter Assays

To investigate the regulatory effect of SOX30 on TNFR2 promoter activity, 293T cell was co‐transfected with the internal control vector pRL‐TK or pcDNA3.1‐SOX30 plasmids, and pGL3 plasmids containing wild‐type or mutated TNFR2 binding sites by lipo2000 Transfection Reagent (Invitrogen) according to the manufacturer's instructions. Following a 48‐h incubation period, dual‐luciferase activity was quantified using the Dual–Luciferase Reporter Assay System (Promega) as previously described [11].

### 4.10 Tissue Microarray Generation and Immunohistochemical (IHC) Analysis

IHC analysis was performed using a SOX30‐specific antibody (1:300; Abcam, ab26024). The expression level of SOX30 was calculated as the product of the classification of positive percentage and the grade of staining intensity. Percentage positivity was categorized as: 0 (< 5%), 1 (5%–25%), 2 (26%–50%), 3 (51%–75%), 4 (≥76%). Staining intensity was graded as: 0 (negative), 1 (weak), 2 (moderate), 3 (strong), and 4 (very strong).

### 4.11 Confocal Immunofluorescent Assay

For Immunofluorescent assay of TNFR2 in MDA‐MB‐231, BT549, and HCC38, cells were fixed in 1% formaldehyde for 15 min and subsequently washed three times with phosphate‐buffered saline (PBS). Following fixation, cells were permeabilized with 0.2% Triton X‐100 for 5 min at room temperature. After three additional PBS washes (5 min each), nonspecific binding sites were blocked by incubating cells with 2% bovine serum albumin (BSA) for 1 h at room temperature. Following another series of three PBS washes (5 min each), cells were incubated with TNFR2 primary antibodies (bsm‐52938R) for 2 h at room temperature. Unbound antibodies were removed through three PBS washes (5 min each). Cells were then incubated with secondary antibodies (1:500 dilution) for 1 h at room temperature in the dark. After the final washing steps (3 × 5 min in PBS), cells were treated with an anti‐fluorescence quencher containing DAPI (4',6‐diamidino‐2‐phenylindole) for nuclear staining. Fluorescent images were captured using a high‐resolution confocal microscope. The Immunofluorescent assay of SOX30, CCL20, and CD206 in lung tissue of patients with breast cancer metastasis was conducted by the above methods. The following primary antibodies were employed in this experiment: SOX30 rabbit polyclonal antibody (abcam, ab272553), CCL20/MIP‐3 alpha Polyclonal antibody (Proteintech, 26527‐1‐AP), and CD206 Polyclonal antibody (Proteintech, 18704‐1‐AP).

### 4.12 Chromatin‐Immunoprecipitation (ChIP)‐PCR Assay

Chromatin‐immunoprecipitation (ChIP) assays were conducted using the ChIP assay kit (Cell Signaling Technology) according to the manufacturer's instructions. Briefly, 5 × 10^6^ MDA‐MB‐231 cells or transfected MDA‐MB‐231 cells were fixed with 1% formaldehyde for chromatin fixation. Following cell lysis, chromatin was digested with micrococcal nuclease to achieve appropriate DNA fragmentation. The digested chromatin was then subjected to immunoprecipitation using specific antibodies. Subsequent steps included analysis of chromatin digestion efficiency, chromatin elution, and DNA purification. Both immunoprecipitated DNA and input control DNA were subsequently subjected to quantitative real‐time PCR (qRT‐PCR) analysis using sequence‐specific primers. The primers were listed as follows. TNFR2‐1 forward primer: GGATAGGGAAACTGGCAGGA; TNFR2‐1 reverse primer: GAAGGCAGGAGGGAGATGTC; TNFR2‐2 forward primer: AAGAGTAGTAACAACAGCCAAGA; TNFR2‐2 reverse primer: CTGGATCTCTGAACTGACCTCA.

### 4.13 NF‐kB TransAM Assay

Cells were collected, and nuclear protein extraction was performed using a nuclear protein extraction kit (Solarbio, EX1470) according to the manufacturer's instructions. NF‐κB p65 activity was assessed using a TransAM NF‐κB p65 kit (Active Motif, Carlsbad, CA, cat. no. 40096), which specifically quantifies the active form of NF‐κB p65. Nuclear extracts were added to the wells of a 96‐well plate pre‐coated with an immobilized oligonucleotide containing the NF‐κB consensus binding site. Active p65 in the nuclear extract binds to the oligonucleotide. Following incubation with specific anti‐p65 antibodies, horseradish peroxidase‐conjugated secondary antibodies were used to generate a sensitive colorimetric readout at 450 nm, measured with a plate reader.

### 4.14 Immune Cell Analysis via CyTOF

The protocol of CyTOF is conducted as described previously [49]. Lung tissues were enzymatically dissociated into single‐cell suspensions using a digestion cocktail containing DNase I, collagenase IV, and hyaluronidase (all from Sigma–Aldrich). After erythrocyte depletion with ACK Lysing Buffer (Sigma–Aldrich), immune cell populations were enriched through percoll density gradient centrifugation. For immunophenotyping, cells were incubated for 30 min at 4°C with a custom‐designed surface marker antibody panel (detailed in Table S1). Following surface staining, cells were fixed overnight at 4°C before undergoing intracellular staining procedures with an intracellular antibody mixture. Mass cytometry analysis was performed on a Helios CyTOF system (Helios, Fluidigm) following the manufacturer's specifications. High‐dimensional data analysis involved the application of t‐distributed stochastic neighbor embedding (t‐SNE) for nonlinear dimensionality reduction, followed by density clustering. The CyTOF experimental procedures and data interpretation were conducted by PLT‐Tech lnc, Hangzhou, china.

### 4.15 Isolation of Primary Macrophages

Using a syringe, aspirate the culture medium and flush it through the mouse bone marrow to collect the cells. Centrifuge at 2000 rpm for 5 min, discard the supernatant, add 1 mL of red blood cell lysis solution, and after 2 min, 2000 rpm centrifugation for 5 min with PBS buffer added. Resuspend the cell precipitate in MEM‐α medium containing 10 ng/ml macrophagecolony stimulating factor (M‐CSF), resuspend the cells, and inoculate them into the petri dishes. After 24 h, aspirate the cells that have not adhered to the dish and inoculate them into new petri dishes.

### 4.16 Flow Cytometry

Lung tissues were harvested, minced into small pieces, and enzymatically digested with collagenase for 1 h at 37°C with gentle agitation. After filtering the digested tissue mixture, centrifuge at 2000 rpm for 5 min, discard the supernatant, then wash the pellet with PBS, followed by repeat centrifugation under identical conditions (2000 rpm, 5 min) and subsequent supernatant removal. Add approximately 1 mL of red blood cell lysis buffer, and incubate for 1–2 min. Filling the tube with PBS and centrifuging at 2000 rpm for 5 min, discard the supernatant, then resuspend the pellet in 200 µL PBS. Add specific antibodies (Alexa Fluor 700 anti‐mouse CD45, Biolegend, 1:200; PE anti‐mouse CD11B, Biolegend,1:200; APC/Cyanine7 anti‐mouse F4/80; Biolegend, 1:200; APC anti‐mouse CD206, Biolegend, 1:200)to each aliquot and incubate at 4°C for 30 min. Filter the cell suspension through a cell strainer into flow cytometry tubes, then analyze the cell counts using flow cytometry.

### 4.17 Mice Models and Treatments

Animal experiments were approved by the Animal Experimentation Ethics Committee of the Third Military Medical University in China. Female athymic Balb/c nude mice and BALB/C mice used in this study aged 5–6 weeks were provided by the Beijing Weitong Lihua Laboratory.

For the nude mouse metastasis model, 1 × 10^6^ SOX30 overexpressed MDA‐MB‐231 cells, SOX30 knockdown HCC38 cells, and control cells were injected into the tail vein of female athymic Balb/c nude mice. After injection for 7 weeks, lung metastases were visualized by in vivo imaging, pulmonary nodule count, and hematoxylin and eosin (HE) staining.

For immune microenvironment analysis, 5 × 10^5^ SOX30 overexpressed and knockdown 4T1 cells placed in PBS were injected into tail vein of female Balb/c mice. Three weeks post‐injection, lung metastases were assessed via in vivo imaging, pulmonary nodule counting, and hematoxylin‐eosin (HE) staining. Additional lung tissues were subjected to flow cytometry analysis.

To deplete macrophages, mice received an intraperitoneal injection of 200 µL clodronate‐loaded liposomes (LIPOSOMA, CP‐002‐002, Netherlands) 24 h before tumor cell inoculation. This initial depletion was followed by maintenance injections of 100 µL clodronate liposomes administered every 72 h for 4 weeks. Control groups received equivalent volumes of empty liposomes following the same administration schedule. For the macrophage rescue experiment, one week after macrophage depletion, macrophages co‐cultured with SOX30‐overexpressing or control cells were injected via the tail vein once a week.

For drug treatment, the following agents were administered intraperitoneally every three days for 3 weeks: anti‐CCL20 monoclonal antibody (R&D; MAB7601; 50 µg each mouse), anti‐mouse TNFR2 (BioXcell; BE0247; 50 µg each mouse), CCR6 inhibitor 1 (MCE; HY‐112701; 10 mg/kg), and Etanercept (MCE; HY‐108847; 10 mg/kg).

### 4.18 Statistical Analysis

Statistical analyses were performed using GraphPad Prism software. Student's t‐test was used to analyze the measurement data. Survival analysis was performed using Kaplan–Meier methods. All graphical representations were generated using GraphPad Prism software. A threshold of *p* < 0.05 was established for determining statistical significance.

## Author Contributions

X.Q., F.H. and Y.Z. conceptualized and designed the work. P.G. performed experiments, analyzed data and wrote the manuscript. N.S. and N.Y. performed animal experiments and analyzed the data, T.Z., R.F., Z.W., Y.Z. and X.T. assist in animal experiments.

## Conflicts of Interest

The authors declared no conflicts of interest.

## Study Approval

Studies involving all mice used in this work followed institutional animal welfare guidelines and legislation, as approved by the laboratory animal welfare and ethics committee of the Army Medical University. The immunological tissue chip used in this study is approved by the ethics committee of Shanghai Outdo Biotech Company. Immunohistochemistry of clinical samples used in this study is approved by the ethics committee of the Southwest Hospital.

## Supporting information




**Supporting File 1**: advs77690‐sup‐0001‐SuppMat.docx.

**Supporting File 2**: advs77690‐sup‐0002‐S1.XLSX.

## Data Availability

The data that support the findings of this study are available from the corresponding author upon reasonable request.
