# Supplementary material for: SOX30 Facilitates Triple‐Negative Breast Cancer Metastasis via TNFR2–NF‐κB Signaling and Tumor Microenvironment Remodeling
Source: Adv Sci (Weinh). 2026 Sep 9:e77690. Online ahead of print. doi: 10.1002/advs.77690 (PMC13558911; doi:10.1002/advs.77690)
Supplement: Supplementary file 1 [file ADVS-9999-e77690-s002.docx]

**Supplementary information**

**SOX30 facilitates triple-negative breast cancer metastasis via TNFR2–****NF-κB signaling and** **tumor microenvironment remodeling**

Pingping Gao1,2#, Na Sun1,2#, Ning Yang^1,2^#, Tingting Zhao^1,2^, Rui Fu^1,2^, Zhenyu Wu^1,2^, Yuqin Zhou^1,2^, Xuanni Tan^1,2^, Yi Zhang^1,2^*, Fei Han^3,4^*, Xiaowei Qi^,2^*

1 Department of Breast and Thyroid Surgery, Southwest Hospital, Army Medical University, Chongqing, 400038, China.

2 Key Laboratory of Chongqing Health Commission for Minimally Invasive and Precise Diagnosis, Chongqing, China

3 Department of Toxicology, School of Public Health, Chongqing Medical University, Chongqing 400016, China.

4 Joint International Research Laboratory of Reproduction and Development of the Ministry of Education, Chongqing 400016, China.

* Corresponding authors: Xiaowei Qi (qxw9908@tmmu.edu.cn), Fei Han (han16897723@163.com), Yi Zhang (yzhang@tmmu.edu.cn)

\# These authors contributed equally to this article.

Conﬂict of Interest: The authors have declared that no conflict of interest exists.

**Figure S1.** The expression and effect of SOX30 on breast cancer cells. (A) The expression levels of SOX30 in breast cancer cells. (B) The relative protein expression levels of SOX30 in MDA-MB-231, BT549, MCF7, and ZR-75-1 cells after overexpression of SOX30. (C) The effect of SOX30 on cell proliferation in MCF7, ZR75-1, MDA-MB-231, and BT549 cells overexpressing SOX30 and in HCC38 and HCC1937 cells with SOX30 knockdown. *: *p*<0.05. **: *p*<0.01.

**Figure S2.** Cell migration and invasion viability of SOX30 on MDA-MB-231 overexpressing SOX30 cells and HCC1937 cells. (A) The protein level of SOX30 in MDA-MB-231 cells overexpressing SOX30, and HCC1937 cells after SOX30 interference. (B) The relative protein expression levels of SOX30 in HCC38 cells after knockdown of SOX30. (C) The migration and invasion viability of SOX30 on MDA-MB-231 overexpressing SOX30 cells. (D) The migration and invasion viability of SOX30 on HCC1937 cells (human TNBC cells). Bar=100 μm. *: *p*<0.05. **: *p*<0.01.

**Figure S3**. SOX30 may promote the metastasis of TNBC through TNFR2. (A) The correlation between SOX30 and TNFR2 expression was analyzed by GEPIA database. (B) Expression of TNFR2 analyzed by CCLE database. (C) Expression of TNFR2 analyzed by TCGA database. (D) The mRNA level of TNFR2 in MDA-MB-231 overexpressed SOX30 cells, BT549 overexpressed SOX30 cells and HCC38 knockdown SOX30 cells. (E) The relative protein expression levels of SOX30 and TNFR2 in MDA-MB-231 and BT549 cells after overexpression of SOX30 or in HCC38 cells after knockdown of SOX30. (F) The binding site of SOX30 and TNFR2 (TNFRSF1B) promoter was predicted by LASAGNA-Search 2.0. *: *p*<0.05. **: *p*<0.01.

 **Figure S4.** SOX30 binds to TNFR2 promoter and co-localizes with TNFR2 in TNBC lung metastasis. (A) The binding of SOX30 on TNFR2 promotor in MDA-MB-231 cells was tested by ChIP assay. (B) SOX30 and TNFR2 immunofluorescence co-localization in lung tissue sections from TNBC patients with lung metastasis. Bar=25 μm. (C&D), IHC staining with SOX30 and TNFR2 in the SOX30-positive and SOX30-negative lung tissues of mice model. Bar=100 μm. **: *p*<0.01.

 **Figure S5.** Quantification of protein expression levels in various cell models. (A) The relative protein expression levels of TNFR2, p65 and p-p65 in MDA-MB-231 and BT549 cells after overexpression of SOX30 or in HCC38 cells after knockdown of SOX30. (B) The relative protein expression levels of TNFR2, p65 and p-p65 in MDA-MB-231 cells overexpressing SOX30 after interfering with TNFR2. *: *p*<0.05. **: *p*<0.01.

**Figure S6**. SOX30 activates NF-κB signaling in breast cancer cells. (A) Representative immunofluorescence images of p65 in MDA-MB-231, BT549, and HCC38 cells overexpressing SOX30. Bar=50 μm. (B) The effects of SOX30 on the transcriptional activity of NF-κB p65 in MDA-MB-231, BT549, and HCC38 cells. (C) The effects of SOX30 on the transcriptional activity of NF-κB p65 in MCF7 and ZR-75-1cells. (D) Protein expression of TNFR2 in MCF7 and ZR-75-1 cells overexpressing SOX30. (E) Effects of NF-κB inhibitor on cell migration and invasion in MDA-MB-231 overexpressing SOX30 cells. *: *p*<0.05. **: *p*<0.01.

**Figure S7.** CyTOF analysis of tumor-infiltrating immune cells. (A) Heatmap of normalized expression for markers expressed in immune cells. The horizontal axis represents the selected marker for design, and the vertical axis represents the automatic clustering of subgroups. (B) Proportion of immune cell subsets in control and SOX30-overexpressin groups.

**Figure S8.** SOX30 promotes M2 polarization of macrophages. (A) Proportion of M2-macrophage after treatment with supernatant from SOX30-overexpressing or SOX30-knockdown cultures, as analyzed by flow cytometry. (B) Proportion of M1-macrophage after treatment with supernatant from SOX30-overexpressing or SOX30-knockdown cultures, as analyzed by flow cytometry. (C) Flow cytometry assessed M2 proportion in macrophages after co-culture with SOX30-overexpressing cells or SOX30-knockdown cells. **: *p*<0.01.

**Figure** **S9**. TAMs educated by SOX30-overexpressing 4T1 cells enhance lung metastasis in vivo. (A) Representative bioluminescent images of BALB/c mice inoculated with 4T1 cells overexpressing SOX30, followed by treatment with control liposomes, clodronate liposomes, or clodronate liposomes for one week, and subsequent injection of tumor-associated macrophages (TAMs) educated by SOX30-overexpressing or control 4T1 cells. Bar=1 cm. (B) Bioluminescence intensity of lung metastasis. (C) Representative images of lung metastasis. (D) Statistical graphs of nodules. (E) H&E staining of lung tissue. Bar=100 μm. **: *p*<0.01.

**Figure S10.** Cell migration and invasion viability of SOX30 on 4T1 cells. (A) The relative protein expression levels of SOX30, TNFR2 p65 and p-p65 in 4T1 cells after overexpression of SOX30 or knockdown of SOX30. (B) The effect of SOX30 overexpression on 4T1 cell migration. (C) The effect of SOX30 overexpression on 4T1 cell invasion. (D) The impact of SOX30 interference on 4T1 cell migration. (E) The impact of SOX30 interference on 4T1 cell invasion. Bar=100 μm.*: *p*<0.05. **: *p*<0.01.

**Figure** **S11**. Multiplex immunofluorescence assay of CK5/6, CD68 CCR6 and CD206 in lung tissue sections from TNBC patients with lung metastasis. Bar=50 μm.

**Figure S12**. M2 macrophage migration assays induced with serum-free medium and 20 ng/mL CCL20 recombinant. Bar=100 μm. **: *p*<0.01.

 **Figure S13.** Immunohistochemical analysis of SOX30, TNFR2, CCL20, CD163, and CD206 in lung metastatic specimens from TNBC patients. *: *p*<0.05. **: *p*<0.01. Bar=50 μm.

**F****igure** **S14**. SOX30 overexpression combined with anti-TNFR2 treatment suppresses lung metastasis in a 4T1 mouse model. (A) Representative bioluminescent images of BALB/c mice inoculated with 4T1 cells overexpressing SOX30 and then administered with anti-TNFR2 neutralizing antibody. Bar=1 cm. (B) Bioluminescence intensity of lung metastasis in control and anti-TNFR2 groups. (C) Images of lung metastasis in control and anti-TNFR2 groups. (D) Statistical graphs of nodules in control and anti-TNFR2 groups. (E) H&E staining of lung tissue in control and anti-TNFR2 groups. Bar=100 μm. *: *p*<0.05. **: *p*<0.01.
